# Anti-Lipoarabinomannan-Specific Salivary IgA as Prognostic Marker for Leprosy Reactions in Patients and Cellular Immunity in Contacts

**DOI:** 10.3389/fimmu.2018.01205

**Published:** 2018-05-30

**Authors:** André Alan Nahas, Mayara Ingrid de Sousa Lima, Isabela Maria Bernardes Goulart, Luiz Ricardo Goulart

**Affiliations:** ^1^Institute of Biotechnology, Federal University of Uberlândia, Uberlândia, MG, Brazil; ^2^Biology Department, Federal University of Maranhão, São Luis, MA, Brazil; ^3^National Reference Center for Sanitary Dermatology and Leprosy, Clinics’ Hospital, School of Medicine, Federal University of Uberlândia, Uberlândia, MG, Brazil; ^4^Department of Medical Microbiology and Immunology, University of California Davis, Davis, CA, United States

**Keywords:** salivary IgA, leprosy reactions, prognostic marker, lipoarabinomannan, household contacts

## Abstract

Leprosy causes the most common peripheral neuropathy of infectious etiology, posing an important public health problem worldwide. Understanding the molecular and immunological mechanisms of nerve damage induced by *M. leprae* is mandatory to develop tools for early diagnosis and preventive measures. The phenolic glycolipid 1 (PGL-1) and lipoarabinomannan (LAM) antigens are major components of the bacterial surface and are implicated on leprosy immunopathogenesis and neural damage. Although the anti-PGL-1 serum IgM is highly used for operational classification of patients, the anti-LAM salivary IgA (sIgA) has not been investigated as diagnostic or prognostic marker in leprosy. Our aim was to assess the presence of anti-LAM sIgA in leprosy patients and their contacts in order to demonstrate whether such expression was associated with leprosy reactions. Distinct patterns of anti-LAM slgA were observed among groups, which were stratified into treatment-naïve patients (116), patients who completed multidrug therapy—MDT (39), household contacts (111), and endemic controls (11). Both anti-LAM sIgA and anti-PGL-I serum IgM presented similar prognostic odds toward leprosy reactions [(odds ratio) OR = 2.33 and 2.78, respectively]. Furthermore, the anti-LAM sIgA was highly correlated with multibacillary (MB) forms (OR = 4.15). Contrarily, among contacts the positive anti-LAM sIgA was highly correlated with those with positive Mitsuda test, suggesting that the presence of anti-LAM slgA may act as an indicator of cellular immunity conferred to contacts. Our data suggest that anti-LAM slgA may be used as a tool to monitor patients undergoing treatment to predict reactional episodes and may also be used in contacts to evaluate their cellular immunity without the need of Mitsuda tests.

## Introduction

Leprosy continues to be the major cause of neuropathies and disabilities worldwide. Despite effective multidrug therapy (MDT), it is still endemic in many regions of the world, especially in Brazil and in India. Most of the infected population remains free of the disease, while a subset of infected individuals develops clinical symptoms, which are associated with the immunity of the host (1). Difficulties persist in clinical conduct, treatment of patients, and monitoring of leprosy reactions, which may lead to nerve damage (2, 3).

Disability in patients with recent diagnosis of leprosy and those who completed MDT treatment continues to be challenging. There is a consensus that the development and installation of neuromotor functional deficiencies and disabilities in leprosy patients are associated with morbidity and chronicity of the disease as pertains to social exclusion and stigma (4).

The major surface antigens of *M. leprae*, lipoarabinomannan (LAM), and phenolic glycolipid 1 (PGL-1), may be detected in saliva, and their participation in mucosal immunity has been under investigation. LAM is exposed on the bacterial surface and is directly implied on the immunopathogenesis of tuberculosis and leprosy (5). The membrane attack complex (MAC) co-localized with LAM in axons has pointed toward the role of this *M. leprae* antigen in the activation of the complement and neural damage in leprosy patients (1, 6).

It has been suggested that IgA may play a role in the protection against infections by mycobacteria of the respiratory tract through the blockage of pathogen entry and/or modulating the pro-inflammatory responses (7). Knockout mice for IgA (−/−) presented greater susceptibility to infection by BCG, compared to normal mice (+/+), as revealed by high bacterial load in the lungs. This result was also followed by an important reduction in IFN-γ and TNF-α in the lungs of IgA (−/−) when compared with IgA (+/+) mice. The detection of antibodies in saliva represents the expression of local immunity (8, 9), but its presence is not sufficient to block the infection process by *M. leprae* (10, 11), although it’s local effect should be considered. Nevertheless, *M. leprae* has been identified in buccal mucosa (12–15).

The presence of salivary IgA (sIgA) against the native LAM antigen in leprosy patients and their contacts has not been investigated yet. Based on prior evidences of the association of LAM with neural damage, and the lack of information of sIgA in patients and contacts, we hypothesized that this response could be used as tool for prognosis of leprosy reactions due to its link with cellular immunity. Therefore, we have performed an investigation on the specific anti-LAM sIgA response and associated outcomes in patients (treatment naïve and treated), contacts and endemic controls, which are discussed herein.

## Materials and Methods

### Studied Population and Group Stratification

Saliva samples were obtained from patients and controls, which were stratified into four groups: group 1: 116 treatment naïve leprosy patients (72 men and 44 women); group 2: 39 leprosy patients (22 men and 17 women) who had completed MDT and were evaluated at discharge (release from treatment), and among them 16 were evaluated at both diagnosis and discharge; group 3: 111 household contacts (40 men and 71 women); and group 4: 11 (11) healthy endemic controls (three men and eight women) were recruited in the population with the following criteria: absence of active leprosy or leprosy in the past, no contact with leprosy patients (family, friend, or colleague), live in the same endemic area, older than 18 years of age, not pregnant or using immunosuppressive medication. All patients and controls were attended at the National Reference Center for Sanitary Dermatology and Leprosy (CREDESH) of the Federal University of Uberlândia (UFU), MG, Brazil, and leprosy reactions were recorded for 3 years, from 2011 to 2014. This study was carried out in accordance with the recommendations of the “Guidelines of the National Board on Human Research Ethics” (CONEP) and with the Declaration of Helsinki, with written informed consent obtained from all subjects. The protocol was approved by UFU Research Ethics Committee under the number 643/11.

### Clinical Data

The operational classification of patients into paucibacillary (PB) and multibacillary (MB) forms were performed for treatment purpose (16), and the clinical classification was done according to Ridley & Jopling (17). Patients’ clinical classification was: 8 tuberculoid (TT); 58 borderline-tuberculoid (BT), in which 29 cases were BT/PB and 29 were BT/MB; 11 borderline–borderline (BB); 17 borderline-lepromatous (BL); and 19 lepromatous form (LL). Additionally, three patients presented the indeterminate form (I).

All patients were submitted to a clinical-laboratorial protocol for the leprosy diagnosis and clinical classification, considering the histopathology of skin lesions, bacilloscopy (18), Mitsuda test results (16, 19), and indirect anti-PGL-1 IgM enzyme-linked immunosorbent assay (ELISA) test (20, 21).

The Mitsuda test was performed on patients to measure the levels of specific cellular immune response for *M. leprae*. Results were obtained 4 weeks after intradermal application of 0.1 mL of the antigen in the right forearm by measurement in millimeters (mm) of the diameter of the local induration. The Mitsuda test results were classified as follows: 0–3 mm—negative; 4–7 mm—weakly positive; 8–10 mm—positive; and greater than 10—strongly positive (16), and previously employed by our group with minor modifications (19), where results were stratified into two categorical groups: “negative” for readings up to 7 mm (0–7 mm), which consisted of negative to weakly positive results, and the “positive” for readings greater than 7 mm (>7 mm), which includes results that are positive and strongly positive with or without ulcerations.

From household contacts, data collection consisted of ELISA anti-PGL-1 serology test and Mitsuda test. The immunization data were assessed according to the presence and number of BCG scars (sBCG 0, 1, or 2 scars). Contacts were further classified according to clinical form (CF) and operational classification of their index case.

### Clinical Characterization of Leprosy Reactions

Leprosy reactions (type 1, type 2, and mixed) were categorized based on clinical and immunological criteria described elsewhere (22). Briefly, type 1 (reversal) reactions occur in the group borderline (BT, BB, and BL) and consisted of acute inflammation in skin lesions or nerves or both. Type 2 reactions occur in LL and BL CFs and cause acute inflammation in any organ or tissue where *M. leprae* are found. Type 2 reactions are also known as erythema nodosum leprosum (ENL). The skin lesions of ENL were characterized by the presence of cutaneous erythematous inflamed nodules and papules that may turn into pustules, then become ulcerated and necrotic. Type 2 reactions often cause neuritis in the form of painful enlarged nerves, nerve function impairment and at systemic level, present high fever, prostration, orchitis, lymphadenopathy, organomegaly, joint involvement, dactylitis, and bone tenderness.

### Saliva Collection

Non-stimulated saliva collection was done by using “Salivette” (Sarstedt, Germany), according to the manufacturer’s instruction. Patients independently collect the sample material using a plain cotton swab. The swab was removed from the Salivette tube and placed in the mouth for chewing for about 60 s to stimulate salivation then the swab was returned with the absorbed saliva to the conical tube. After centrifugation at 5,000 rpm at 4°C for 10 min, a clear saliva sample was obtained, aliquoted, transferred to 0.5 mL microtubes, and frozen at −20°C. Sample volumes varied from 0.5 to 1.5 mL.

### Indirect ELISA for Detection of Anti-LAM Salivary IgA and Anti-PGL-1 Serum IgM

High affinity plates (Maxsorp—Nunc^®^) with 96 wells were sensitized with 50 µL of native LAM (BEI RESOURCES)^1^ diluted in carbonate/bicarbonate buffer (50 µL of native LAM 100 µg/mL diluted in 4,950 µL of carbonate/bicarbonate buffer, pH 9.6). The plates were incubated overnight in a cold chamber at 4°C. Four washings were done with PBS_T_ 0.05% (200 μL/well) and saliva samples diluted in 5% PBS/BSA (1:5) were added in triplicate. The plates were incubated for 1 h at 37°C, and after five washings with PBS_T_ 0.05%, 50 µL of anti-IgA were added (CALBIOCHEM^®^, USA; 1.0 mg/mL) labeled with diluted peroxidase 1:1,000 in PBS/BSA and incubated for 1 h at 37°C. After six washings with PBS_T_ 0.05%, reactions were developed by adding 50 µL of OPD solution for 5 min (2 mg OPD + 5,000 µL citrate buffer + 2 µL H_2_O_2_), and the reaction was then stopped with 20 μL/well of sulfuric acid (H_2_SO_4_ 2N). ELISA readings were performed in a microplate reader (TP—READER, THERMO PLATE) at 492 nm.

The anti-PGL-1 ELISA was also an indirect test to detect circulating IgM antibodies in serum against the *M. leprae* native PGL-1, and it was performed as previously described (23).

### ELISA Index (EI)

Saliva samples were processed in triplicate. Results were converted into an EI, in which a value of 1.1 was considered a positive threshold. For the EI calculation, the absorbance mean value was divided by the cutoff, considering the values greater than 1 as positive. The cutoff value was obtained with absorbance readings of negative controls, and three SDs were added to the mean (23).

### Statistical Analysis

A descriptive analysis was used for all patients, contacts, and controls. The normality of samples was verified by the Shapiro–Wilk test. The variables did not present normal distribution. The Mann–Whitney *U* and Kruskal–Wallis tests were performed to test whether medians between groups were different, under the assumption that the shapes of the underlying distributions were the same. The non-parametric tests were performed with GraphPad Prism software version 5.0 (GraphPad Software, San Diego, CA, USA), and the odds ratios (OR) were calculated through the MedCalc server.^2^ Significant values were considered when *P* ≤ 0.05.

## Results

The frequency distribution of treatment naïve leprosy patients with or without reactions (during or soon after MDT treatment) was stratified according to the operational classification, gender, Mitsuda test, ELISAs anti-LAM sIgA, and anti-PGL-1 IgM (Table 1). Patients with MB leprosy presented higher chances of developing leprosy reactions (OR = 4.15; *P* < 0.001) without gender preference. A significant positive correlation was observed between anti-LAM slgA+ and leprosy reactions. Among reactional patients, 69.4% (34/49) were also anti-LAM positive at diagnosis, with a 2.33-fold higher chance of developing reactions. Similarly, the positive IgM serology also showed a significant correlation with leprosy reactions (OR = 2.78; *P* < 0.008).

**Table 1:** Frequencies of treatment naïve leprosy patients with or without reactions during or after MDT, divided according to their operational classification, gender, Mitsuda test result, anti-phenolic glycolipid 1 (PGL-1) IgM serology, and anti-lipoarabinomannan (LAM) sIgA in saliva, obtained at diagnosis.

Variables			Leprosy reactions			Odds ratio	Confidence interval (95%)	*P*
			Yes		Total (*n*)			
			*n*	(%)				
**Operational classification**								
Multibacillary (MB)			41	(52.6)	78	**4.15**	**1.69–10.19**	**0.001**
Paucibacillary (PB)			8	(21.1)	38			
**Gender/operational classification**								
Male	72	MB	24	(45.3)	53	**4.41**	**1.14–16.96**	**0.030**
		PB	3	(15.8)	19			
Female	44	MB	17	(68)	25	**5.95**	**1.58–22.32**	**0.008**
		PB	5	(26.3)	19			
Total			49	(42.2)	116			
**Mitsuda**								
0–7 mm			16	(35.6)	45	2.34	0.67–8.17	0.181
>7 mm			4	(19.0)	21			
**Anti-PGL-1 IgM**								
PGL-1+			32	(54.2)	59	**2.78**	**1.29–5.98**	**0.008**
PGL-1−			17	(29.8)	57			
**Anti-LAM sIgA**								
LAM+			34	(50.7)	67	**2.33**	**1.07–5.06**	**0.031**
LAM−			15	(30.6)	49			

*Mitsuda test reading system: “negative” for readings up to 7 mm (0–7 mm) and “positive” for readings greater than 7 mm (>7 mm) (17). P, probability value*.

*Bold fonts were used to emphasize the significant values*.

The frequency distribution of leprosy patients by CF, type of leprosy reactions, and positivity for secretory anti-LAM IgA is shown in Table 2. The BT form was the only form that presented significant correlation with type 1 leprosy reaction, with a 6.9-fold higher chance of having reactions (*P* < 0.006). In group 1, 12 patients (10.3%) were household contacts that became ill (6 BT/PB; 3 BT/MB; 1 I/PB; 1 TT/PB; 1 BL/MB), and among them, one developed a type 1 reaction after discharge (female, BT/MB with positive salivary anti-LAM sIgA at the time of diagnosis). Regarding the small ORs for the MB forms (BB, BL, and LL), it is important to emphasize that the sample size collected for these forms was very small, so data should be carefully interpreted. Our data corroborate the notion that salivary sIgA+ is associated with type 1 (reversal) reaction, since only PB CFs presented very large odds, followed by small ORs with lack of significance in MB forms.

**Table 2:** Frequencies of leprosy patients by CF, type of leprosy reactions, and positivity for salivary anti-lipoarabinomannan (LAM) sIgA, followed by odds ratios (OR), confidence interval at 95% (CI_95%_), and probability levels (P) toward the occurrence of reactions.

CF	n	Anti-LAM sIgA	Total, n(%)	Reaction (n)			Total (n)		OR	Confidence interval (95%)	P
				T1	T2	M	R	No			
TT	8	+	4 (50)	2	0	0	2	2	9.00	0.29–271.67	0.20
		−	4 (50)	0	0	0	0	4			
BT	58	+	33 (56.9)	16	0	0	16	17	**6.90^a^**	**1.72–27.60**	**0.006**
		−	25 (43.1)	3	0	0	3	22			
BB	11	+	7 (63.6)	3	0	0	3	4	0.08	0.003–2.20	0.138
		−	4 (36.4)	4	0	0	4	0			
BL	17	+	10 (58.8)	2	1	3	6	4	1.12	0.15–7.98	0.906
		−	7 (41.2)	3	0	1	4	3			
LL	19	+	12 (63.2)	0	6	1	7	5	1.05	0.15–6.92	0.959
		−	7 (36.8)	0	4	0	4	3			
Total	116			33	11	5	49	67			

*Bold fonts were used to emphasize the significant values*.

*^a^Statistically significant*.

*CF, clinical form; TT, tuberculoid; BT, borderline-tuberculoid; BB, borderline–borderline; BL, borderline-lepromatous; LL, lepromatous*.

*Reaction (R): T1 = reaction type 1 (RR, reversal reaction); T2 = reaction type 2 (ENR, erythema nodosum reaction); M = mixed reaction (T1/T2); No = no reaction*.

Thirty-nine patients were evaluated at the time of discharge from MDT, in which 35.9% (14/39) presented positive salivary anti-LAM slgA. Among them, 16 patients were also evaluated at diagnosis, in which 9 were males and 7 females, 10 MB and 6 PB. In this group, nine patients (56.2%) developed leprosy reactions, in which one was PB and eight were MB. At discharge from MDT, the mean EI anti-LAM slgA of patients with reaction was 0.91, while patients without reactions presented an EI mean of 0.53 (*p* = 0.29). Considering the stratification of patients’ groups into those with reactions and without reactions, the EI kinetics from diagnosis (D) to discharge (A) displayed a different profile, in which the group without reaction had decreasing values or remained low (data not shown).

**Figure 1:**
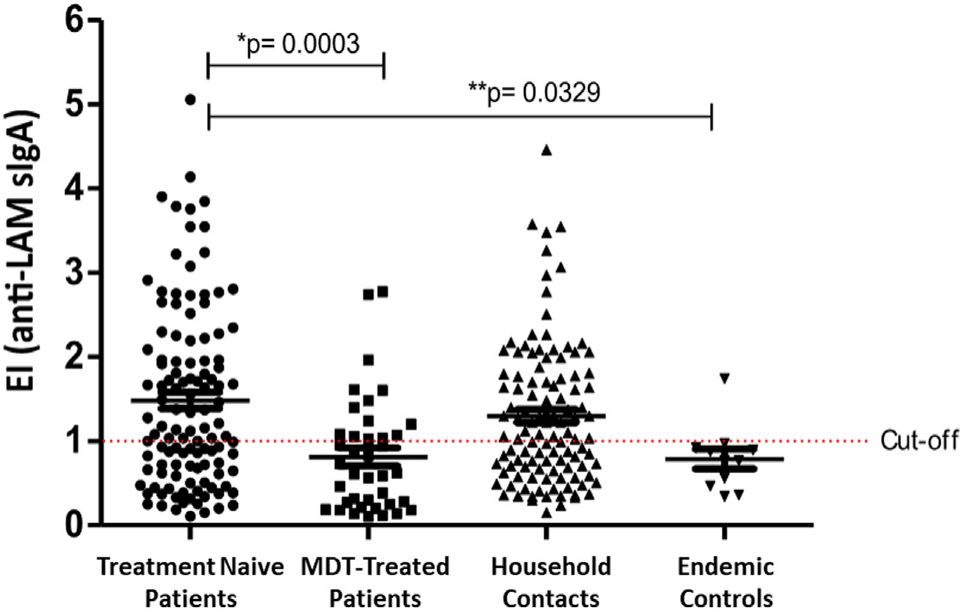
Enzyme-linked immunosorbent assay (ELISA) index values of salivary anti-lipoarabinomannan (LAM) sIgA detection in patients at diagnosis, patients at MDT discharge, household contacts, and endemic controls. Cutoff value of ELISA index is ≥1. Median comparisons performed with the Kruskal–Wallis test.

Table 3 shows the ELISA results using salivary anti-LAM sIgA from 111 contacts, which were correlated with the Mitsuda test, and the presence/absence of the sBCG. For the Mitsuda test, values of 0–3 mm (0) were considered as the worst prognosis (−), and values ≥4 as the best prognosis (+). For sBCG, negative (−) was considered as absence of scar (0) and positive (+) was considered with the presence of 1 or 2 scars (Table 3). There was a positive correlation between anti-LAM slgA+ and positive Mitsuda test, with a significant OR (OR = 0.29; *p* = 0.011), suggesting that positivity for anti-LAM in the saliva of contacts may be an indicator of natural resistance to leprosy, due to the greater frequency of positive sIgA in Mitsuda-positive individuals (OR = 3.41; *p* = 0.011). Significant differences of salivary sIgA were observed among patients between groups 1 and 4 (*p* = 0.0329) and between groups 1 and 2 (*p* = 0.0003) (Figure 1), suggesting that treatment reduces the bacillary load, which is reflected by detecting reduced anti-LAM sIgA in saliva in most patients, except in those that presented leprosy reactions. The Figure S1 in Supplementary Material is presented with raw ELISA data in saliva to demonstrate the range of original values found in each group before transformation to ELISA indices.

**Table 3:** Prognostic analyses through odds ratio (OR) calculations for household contacts considering the interactions of the three prognostic factors: Mitsuda test, presence of BCG scar (sBCG), and salivary anti-lipoarabinomannan (LAM) sIgA.

Markers interactions		sBCG*		OR	Confidence interval (95%)	P
		−	+			
Salivary IgA	+	14	47	1.56	0.59–4.09	0.362
	−	8	42			
	
		Mitsuda*				
		−	+			
	+	8	53	**0.29**	**0.11–0.75**	**0.011**
	−	17	33			
	
		Mitsuda/sBCG*				
		−	+			
	+	2	41	0.29	0.05–1.61	0.15
	−	5	30			

*Bold fonts were used to emphasize the significant values*.

*sBCG (−) = worse prognosis (0 cBCG) and (+) = better prognosis (1 and 2 sBCG)*.

*Mitsuda (−) = worse prognosis (0–3 mm) and (+) = better prognosis (≥4 mm)*.

*Mitsuda/sBCG (–) = worse prognosis (Mit < 4/sBCG-ID = 0) and (+) = better prognosis (Mitsuda ≥ 4/sBCG ≥ 1)*.

Among contacts that presented 0 (no scar), 1, and 2 sBCGs, 63.6% (14/22), 51.4% (37/72), and 58.8% (10/17) were positive for salivary LAM, respectively. Among those without sBCG, a significant positive association was observed between sIgA+ and positive Mitsuda test (OR = 10.00; CI_95%=_1.26–79.3; *p* = 0.02). Contacts without BCG scar were vaccinated with BCG as soon as they entered the CREDESH’s monitoring program.

The endemic control (EC) group consisted of 11 volunteers without any personal or familial history of leprosy, and the very small sample size in this group may seem a weakness of the paper if one considers the marker for diagnostics, which is not the case. The aim was to demonstrate the validity of the anti-LAM sIgA as a prognostic marker in patients and contacts, so endemic controls did not contribute for the calculation of odds, although it provided additional support to the data, since only one individual presented a positive ELISA. It is possible that this individual has been exposed to *M. leprae* through a contact or patient without knowing this condition.

## Discussion

The present study characterized the salivary anti-LAM secretory IgA response in leprosy patients and their contacts and suggests its use as a prognostic tool for leprosy reactions in patients, and as immunity status in contacts by associating sIgA values with laboratorial and clinical parameters. Saliva has been the study subject as diagnostic or as supplementary tool for diagnosis or for monitoring of oral and systemic diseases (24, 25). The simple sample collection, the minimum invasiveness, and diminished contamination risk for healthcare professionals represent important aspects to support the choice of saliva as a promising tool for diagnosis and for monitoring of clinical evolution of patients during treatment and post-discharged.

Besides being the primary surface antigen and one of the dominant virulence factors of *M. leprae*, LAM also shows a close relationship with leprosy reactions, since it has promoted neural damage in a mouse model by activating the complement system *via* MAC (1, 6). Our data corroborate this immune response by showing a positive correlation between positive anti-LAM slgA with the occurrence of leprosy reactions, suggesting the involvement of exacerbated cellular response against LAM of *M. leprae*. Our results are also supported by the evidence that deposition of complement is associated with LAM of *M. leprae* in leprosy lesions, and positivity for LAM in the nerves is also associated with deposition of MAC (1). Additional support for the involvement of LAM with the occurrence of leprosy reactions comes from the fact that even after finishing treatment, LAM can still be detected in skin and nerve biopsies, with a clearance that is even slower than that of PGL-1 (26).

Our data also point out toward a greater occurrence of leprosy reactions in patients with MB leprosy, which are also corroborated by other studies elsewhere (3, 27), and interestingly, we also showed that this association is highly linked with detection of anti-LAM salivary sIgA. Patients with MB leprosy and with positive anti-LAM slgA presented chances fourfold higher toward having leprosy reactions than those with negative results. Nevertheless, considering the positivity of anti-LAM salivary sIgA in all patients, the chances are at least twofold greater toward the development of leprosy reactions.

The distinctive behavior of salivary anti-LAM slgA, especially when we compared patients with endemic controls, suggests that salivary anti-LAM can be a marker of exposure to *M. leprae*. This information is even strengthened when patients groups (1 and 2) are compared, in which treated patients (group 2) displayed a significant lower positivity for anti-LAM slgA than that of the treatment naïve patients (group 1), suggesting that MDT monitoring with this marker is possible. The differences in immune salivary response between the contact and the endemic controls reinforces the role of anti-LAM IgA as an indicator of exposure to *M. leprae*, as proposed elsewhere (28). Interestingly, we have also evaluated patients for anti-LAM slgA both at diagnosis and at discharge from MDT, and those who maintained or presented elevated their levels during treatment had greater chances of developing leprosy reactions than those whose levels of anti-LAM slgA had declined, further supporting the results obtained for all leprosy patients, and demonstrating the importance of anti-LAM slgA as a predictive biomarker of leprosy reactions in patients.

Our results with contacts also demonstrated that anti-LAM slgA significantly correlated with the positive Mitsuda test, suggesting that positivity for anti-LAM in saliva may be used as an indicator of resistance to leprosy, either conferred by prior exposure or by natural resistance. These results are corroborated elsewhere, in which IgG positivity to LAM was significantly increased in patients vaccinated with BCG and in patients with active tuberculosis. Oral vaccination with BCG induced a significant increase of secretory IgA to LAM as well. These authors suggested that trials with immunoglobulins reactive to LAM may serve as markers of humoral and cellular response in future vaccinations with BCG and/or with attenuated mycobacteria (29). These results were also corroborated in another study that showed significant increases in specific anti-LAM IgGs after primary vaccinations and in booster doses of BCG (30). Although prior exposure to *M. leprae* can also lead to humoral and cellular responses, it is likely that the presence of sIgA in the saliva of household contacts may also suggest oral immunization, correlating with a cellular response characterized by the positive Mitsuda test, performed before the saliva collection during the first analysis of the contact. Prior studies have indicated generalized subclinical transmission of *M. leprae* with transient infection of the nose, and possibly in the oral cavity (29), resulting in the development of a mucosal immune response that can be protective (31). It remains to be verified whether contacts with negative anti-LAM slgA in saliva along with other parameters, such as absence of BCG scar and presence of serum PGL-1, represent a greater risk of disease development.

Our data indicate that there is no benefit in testing individuals of unknown leprosy status, because salivary anti-LAM sIgA cannot be used as a diagnostics marker, due to its absence in more than 40% of patients at diagnosis and persistence of detection in more than 35% of patients at discharge. However, monitoring anti-LAM slgA in saliva of leprosy patients undergoing treatment may become an important tool in detecting groups at risk for the development of leprosy reactions, especially type 1, and positivity in household contacts suggests greater resistance to leprosy; however, the possibility of resistance to exposure to *M. leprae* should be further investigated. Importantly, the worldwide absence of Mitsuda tests to evaluate contacts and patients’ cellular immunity poses an important issue in leprosy monitoring programs, in which the salivary anti-LAM slgA may become a good substitute tool of the Mitsuda test due to its high correlation with it. Besides, saliva avoids de use of this invasive procedure with intradermal injection of standardized extract of inactivated bacilli, and ELISA takes just a few hours instead of 21 days for reaction evaluation.

## Ethics Statement

This study was carried out in accordance with the recommendations of “Guidelines of the National Board on Research Ethics (CONEP)” with written informed consent from all subjects. All subjects gave written informed consent in accordance with the Declaration of Helsinki. The protocol was approved by UFU Research Ethics Committee/CEP under the number 643/11.

## Author Contributions

LG: senior author, conceived the concept and the experimental design, performed biological assays, statistical analyses, manuscript writing and revision. IG: co-senior author, sample collection, data interpretation, manuscript writing and revision. AN: performed biological assays, statistical analyses and interpretation, and manuscript writing. ML: performed biological assays and manuscript writing.

## Conflict of Interest Statement

The authors declare that the research was conducted in the absence of any commercial or financial relationships that could be construed as a potential conflict of interest.

## Footnotes

^1^https://www.beiresources.org/ (Accessed: May 19, 2018).

^2^www.medcalc.org/calc/odds_ratio.php (Accessed: May 19, 2018).

## Supplementary Material

Figure S1 Enzyme-linked immunosorbent assay values (absorbances at 492 nm) of anti-lipoarabinomannan (LAM) sIgA detection in patients at diagnosis, patients at MDT discharge, household contacts, and endemic controls. Median comparisons performed with the Kruskal–Wallis test.
