# Supplementary figures and images for: Anti-Lipoarabinomannan-Specific Salivary IgA as Prognostic Marker for Leprosy Reactions in Patients and Cellular Immunity in Contacts
Source: Front Immunol. 2018 May 30;9:1205. doi: 10.3389/fimmu.2018.01205 (PMC5990868; doi:10.3389/fimmu.2018.01205)

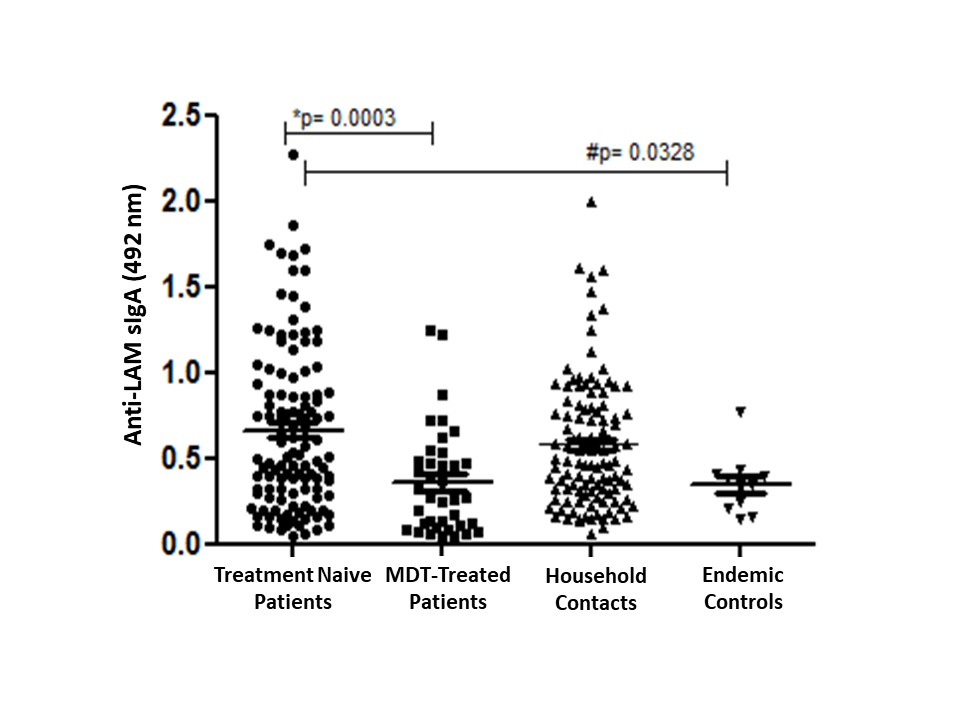

Supplement: Figure S1 — Enzyme-linked immunosorbent assay values (absorbances at 492 nm) of anti-lipoarabinomannan (LAM) sIgA detection in patients at diagnosis, patients at MDT discharge, household contacts, and endemic controls. Median comparisons performed with the Kruskal–Wallis test. [file Image_1.tif]
